# Association Between Swallowing Dysfunction and Multidimensional Quality of Life Among Community-Dwelling Healthy Korean Older Adults: A Pilot Cross-Sectional Study

**DOI:** 10.3390/healthcare13222964

**Published:** 2025-11-19

**Authors:** Hyun-Ah Jang, Jun-Seon Choi

**Affiliations:** 1 Department of Health Science, Graduate School of Gachon University, 191 Hambakmoero, Yeonsu-gu, Incheon 21936, Republic of Korea; 202050531@gachon.ac.kr; 2 Department of Dental Hygiene, College of Medical Science, Gachon University, 191 Hambakmoero, Yeonsu-gu, Incheon 21936, Republic of Korea

**Keywords:** swallowing dysfunction, dysphagia, quality of life, CASP-19 scale, older adults

## Abstract

**Background:** As life expectancy is increasing, the quality of life of older adults has become critical. Health status of older adults is a significant predictor of quality of life, making it essential to consider the diseases and conditions prevalent among this population. Although swallowing dysfunction is an emerging health problem, even in healthy older adults, few studies have examined the impact of these disorders on the quality of life. Therefore, in this pilot cross-sectional study, we aimed to examine the association between swallowing dysfunction and quality of life among 105 community-dwelling healthy older adults with no history of diseases that may cause swallowing dysfunction and who were screened normal on a dementia test. **Methods:** Quality of life was assessed using the CASP-19 scale. The risk of swallowing dysfunction was assessed using two screening tools, namely the Dysphagia Risk Assessment Scale and the Repetitive Saliva Swallowing Test. The strength of the association between swallowing dysfunction and quality of life was analyzed using independent *t*-tests, one-way analysis of variance, Kruskal–Wallis test, and multiple linear regression. **Results:** At least one in three participants had a high risk of swallowing dysfunction. The multiple linear regression model adjusted for several factors associated with the quality of life of older adults revealed that the high-risk group for swallowing dysfunction had a lower quality of life than the control group (β = −0.179 for Model 1; β = −0.201 for Model 2, *p* < 0.05). **Conclusions:** This study identified a potential association between swallowing dysfunction and lower quality of life. The findings suggest that maintaining or improving swallowing function may be a valuable component of strategies aimed at enhancing overall quality of life in healthy older adults.

## 1. Introduction

The United Nations, in its “World Population Prospects 2022” report, projected that the global population aged ≥65 years will more than double in the next 30 years compared to that in 2020 [1]. In particular, the aging population in South Korea is increasing faster than in any other country in the world. It became an aged society in 2018, with the number of individuals aged ≥65 years exceeding 14% of the total population, and is set to become a super-aged society in 2025, with the number increasing to 20% [2]. As a result, interest in older adulthood is increasing, particularly in understanding how this population can lead a happy and meaningful life during this stage.

Quality of life is a subjective state of well-being that includes happiness, satisfaction, and positive emotions [3]. Older adults undergo various physical, mental, and social changes during aging and are easily exposed to risk factors that can impair their quality of life [4]. Quality of life is a key indicator of the overall status of life in older adults [4]. Furthermore, the quality of life of older adults deserves more attention because it can help predict serious geriatric problems such as depression, which can increase suicide risk [5,6], and it is associated with active and successful aging—concepts that have recently received a lot of attention [7].

The quality of life of older adults is determined by a complex interplay between various factors [8]. However, health status, including depression, dementia, pain, and oral health, significantly impacts the quality of life in older adults [9,10,11]. Therefore, health-centered interventions can play a positive role in improving their quality of life. In particular, oral health is closely related to overall well-being and successful aging [12] because the oral cavity, the first organ of the digestive system, plays a vital role in mastication, pronunciation, and swallowing in coordination with other organs such as the teeth, tongue, and lips [13]. Older adults with ≥20 teeth are less likely to experience health decline and have a higher quality of life than those with fewer teeth [14]. Fewer remaining teeth and accelerated tooth loss are associated with social isolation, which impairs the overall well-being and quality of life [15]. In addition, swallowing dysfunction, in which regurgitation or delayed passage of food and liquid bolus occurs because of abnormalities in the muscles or nerves involved in deglutition, may have negative socioemotional effects in older adults [16]. Swallowing dysfunction or dysphagia is caused by several medical conditions, including neurological diseases, and is not a disease in itself [17]. Choking and coughing during or immediately after eating, which are common symptoms of swallowing dysfunction, can prolong meal duration and create a fear of eating [16,18]. Particularly, patients with swallowing dysfunction who exhibit difficulties in eating may experience depression, anxiety, and social isolation, which may ultimately affect the quality of life [19]. Additionally, prolonged swallowing dysfunction can result in further deterioration of health or increase mortality owing to its significant negative outcomes such as malnutrition, dehydration, weight loss, and aspiration pneumonia [18,20].

Although the incidence of swallowing dysfunction is reportedly high in patients with neurological diseases [21], oropharyngeal dysphagia in particular also has a notably high incidence in healthy older adults [17]. A recent systematic review and meta-analysis estimated that one in three community-dwelling older adults and nearly half of older adults experience swallowing dysfunction [22]. Lee et al. [23] reported that approximately 39% of healthy community-dwelling older adults have swallowing problems. Another study using the water-swallowing test found that approximately 35% of healthy older women without neurological diseases had swallowing dysfunction, yet only a few reported these symptoms on swallowing function questionnaires [24]. The high incidence of swallowing dysfunction is explained by the fact that various muscles and structures involved in swallowing, including the oral cavity and pharynx, degenerate or lose flexibility with age, diminishing the chewing function and swallowing efficiency [18]. Sarcopenia directly reduces the strength of swallowing muscles and impairs oropharyngeal function [25]. These findings indicate that swallowing dysfunction should be recognized as a common disorder in aging societies. Additionally, greater social attention is needed to address the challenges faced by healthy older adults because of swallowing dysfunction, particularly its negative impact on well-being, which has not been adequately addressed.

Most studies on swallowing dysfunction have focused on its prevalence in specific populations, diagnostic methods, risk factors, and rehabilitation methods. Though the negative impact of swallowing dysfunction on the quality of life has been reported in individuals with diseases that may cause swallowing dysfunction, such as stroke and Parkinson’s disease [26,27,28,29,30], the impact may have resulted from the underlying etiology. Given the relatively high prevalence of swallowing dysfunction in healthy older adults and the tendency of some individuals to perceive these disorders as a normal part of aging, leading them not to report symptoms to healthcare providers or seek appropriate treatment [24,31,32], there is a need to examine the negative outcomes of these disorders in terms of psychosocial well-being and life satisfaction. To date, few studies have investigated the relationship between swallowing dysfunction and the quality of life from a multidimensional perspective in healthy older adults. Therefore, this study analyzed the association between swallowing dysfunction and quality of life among healthy community-dwelling Korean older adults who had no disease known to contribute to swallowing dysfunction and were screened as cognitively normal.

## 2. Materials and Methods

### 2.1. Participants

**Figure 1:**
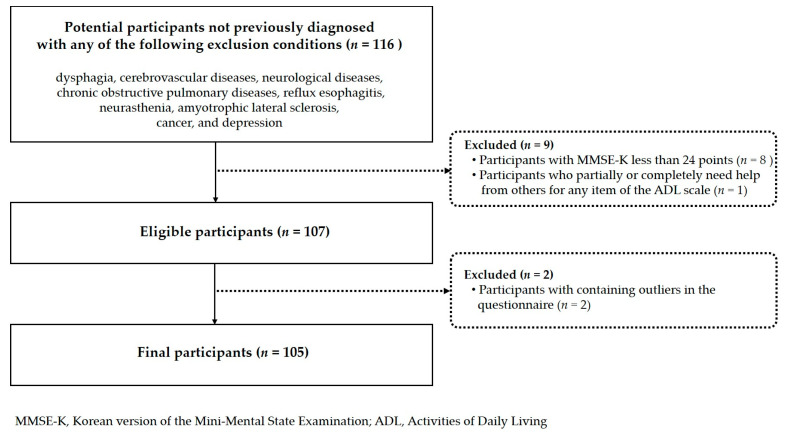
Flowchart of participant recruitment process.

This pilot cross-sectional study was approved by the Institutional Review Board of Gachon University (No. 1044396-202308-HR-154-01) and conducted in compliance with the Declaration of Helsinki of the World Medical Association. The purpose and procedures of the study were fully explained to individuals aged ≥65 years who visited senior welfare facilities in the Incheon and Gyeonggi Provinces and were selected through convenience sampling. In this study, to minimize the influence of disease on quality of life, individuals with any conditions known to be associated with swallowing dysfunction were excluded, based on criteria from previous studies [33,34]. These conditions included dysphagia, cerebrovascular diseases such as cerebral infarction, neurological diseases such as Parkinson’s or Alzheimer’s disease, chronic obstructive pulmonary diseases including chronic bronchitis, reflux esophagitis, neurasthenia, amyotrophic lateral sclerosis, cancer, and depression. Two preliminary tests using different scales were conducted on older adults willing to participate in this study: the Mini-Mental State Examination-Korean version (MMSE-K) scale to screen for cognitive impairment [35] and the Korean Activities of Daily Living (ADL) scale to screen for functional impairment [36] (*n* = 116). Individuals with an MMSE-K score lower than 24 [35] (*n* = 8) and individuals who reported receiving partial or full assistance from others in at least one of the seven activities of daily living (ADL) [37] were excluded from the study (*n* = 1). Informed consent was obtained from 107 participants who understood the study procedures and methods and voluntarily agreed to participate in the study. The participant recruitment process is illustrated in Figure 1. Data were collected through face-to-face interviews from 22 November 2023, to 31 May 2025. After data collection, data pertaining to two questionnaires containing outliers were excluded; therefore, data from 105 participants were analyzed in this study. The participant age ranged from 65 to 90 years, with a mean age of 74.50 ± 6.81 years. The minimum sample size for multivariate linear regression was calculated as 92 using G^*^ Power 3.1.9.4 (Heinrich-Heine-University Düsseldorf, Düsseldorf, Germany) with the following parameters: 80% power, 5% significance level, 0.15 effect size, and five predictors [38].

### 2.2. Measurements

In this study, data pertaining to sociodemographic characteristics (sex and four others), health behaviors (health check-up results within the past two years and nine others), oral and general health status (self-perceived general health and ten others), swallowing dysfunction (Dysphagia Risk Assessment Scale [DRAS] and Repetitive Saliva Swallowing Test [RSST]), and the overall quality of life, were collected by a single trained examiner.

To objectively evaluate chewing ability, the participants were instructed to chew a color-changeable chewing gum (Masticatory Performance Evaluating Gum XYLITON; Lotte Co., Tokyo, Japan), which changes from yellow-green to red [39]. It contains xylitol, citric acid, and various dyes, and the pH-sensitive red dye changes color when exposed to a neutral or alkaline environment [40]. The participants were instructed to chew the gum directly for 2 min and then spit it on a disposable dental mixing pad. The chewed gum was flattened to a thickness of approximately 1.5 mm with a spatula, and its color was compared with the manufacturer’s evaluation index to assess the chewing ability. The scores ranged from 1 to 10, with higher scores indicating a better chewing ability [40]. Frailty status was assessed using the FRAIL scale developed by Morley et al. [41], which consists of five items: fatigue, resistance, ambulation, illness, and loss of weight. Total scores range from 0 to 5 and were categorized as normal (0), pre-frailty (1–2), or frailty (≥3) [42]. The DRAS developed by Fukada et al. [42] was used to identify community-dwelling older adults at risk of swallowing dysfunction. It consists of 23 items and the total score ranges from 0 to 69, with higher scores indicating a greater risk [42]. Individuals with a score of ≥6 were classified into the high-risk group for swallowing dysfunction [29,43]. Given that most older adults were unaware of or did not report swallowing dysfunction to health professionals [24,32], the RSST, a simple noninvasive screening test that can objectively evaluate the decline in swallowing function, was also administered [44]. The examiner palpated the prominentia laryngea of each participant and recorded the number of times the participant swallowed saliva for 30 s. Those who swallowed fewer than three times were classified into the high-risk group for swallowing dysfunction [44]. Finally, considering the WHO definition of quality of life, the CASP-19 scale, a multidimensional instrument, was employed to measure the broader dimensions of quality of life [45]. This tool is specifically designed to assess the quality of life in older adults and has good reliability and validity [46,47]. It consists of 19 items across four domains: control, autonomy, self-realization, and pleasure [45,46]. Each item was rated from never (0) to often (3), with total scores ranging from 0 to 57 [45]. Higher scores indicate a better quality of life [45].

### 2.3. Statistical Analysis

The collected data were analyzed using SPSS (IBM SPSS Statistics 23.0, IBM Corp., Armonk, NY, USA). To analyze the factors associated with the quality of life in older adults, independent *t*-tests, one-way analysis of variance (ANOVA) followed by Scheffe’s post hoc test, and the Kruskal–Wallis test were conducted. Post hoc analysis for the Kruskal–Wallis test was performed using the Mann–Whitney U test with Bonferroni correction. Additionally, stepwise multivariate linear regression was performed to identify the impact of swallowing dysfunction on the quality of life of older adults. In the final regression model, the dependent variable was the quality of life, while the independent variables included all factors with *p <* 0.05 in the respective univariate analyses. The significance level was set at *p* < 0.05.

## 3. Results

### 3.1. Quality of Life by Socio-Demographic Characteristics

The mean value of quality of life (assessed using CASP-19) of the study participants was 35.76 (±9.94). Older adults who perceived their economic status as high reported a higher quality of life than did those who perceived it as moderate (*p* < 0.05). Although participants with a Christian religion had a higher quality of life than their respective control groups, the differences were not statistically significant (*p* = 0.069) (Table 1).

### 3.2. Quality of Life by Health Behaviors

The quality of life was higher among older adults who engaged in moderate-intensity exercise (e.g., ≥10 min of daily brisk walking), brushed their teeth ≥ 3 times a day, and underwent regular oral check-ups and scaling, than among controls (*p* < 0.05) (Table 2).

### 3.3. Quality of Life by Oral and General Health Status

Quality of life was higher among older adults who perceived their overall health as good, had no more than one systemic disease, reported very low stress, or were classified as robust rather than pre-frail on the frailty screening test (*p* < 0.05). It was also higher among those with ≥ 26 remaining teeth, no need for maxillary dental prostheses, and excellent chewing ability (score ≥ 9) than among controls (*p* < 0.05). Patients with a normal swallowing ability assessed using DRAS and RSST also exhibited a higher quality of life than did the high-risk group for swallowing dysfunction (*p* < 0.05). Although those who did not require mandibular dental prostheses tended to have a higher quality of life, the difference was not statistically significant (*p* = 0.079) (Table 3).

### 3.4. Intensity of Association Between Swallowing Dysfunction Risk and Quality of Life

The results of stepwise multiple linear regression, which analyzed the intensity of the association between quality of life and swallowing dysfunction risk in older adults, are presented in Table 4. Those with a high economic status (β = 0.240), who were Christian (β = 0.226), and who brushed their teeth more frequently (β = 0.223) had a higher quality of life. Conversely, those who did not engage in moderate-intensity exercise (β = −0.190) or periodic scaling (β = −0.187), had higher perceived stress levels (β = −0.206), and were at high risk of swallowing dysfunction (DRAS score ≥ 6) (β = −0.179) tended to have a lower quality of life (*p* < 0.05) (Model 1). When the high-risk group for swallowing dysfunction assessed by the DRAS in the regression model was replaced with the high-risk group assessed by the RSST (Model 2), the significant independent variables remained the same as in Model 1, and high risk of swallowing dysfunction (RSST times ≤ 2) continued to be associated with a lower quality of life (β = −0.201, *p* = 0.022). Residual analyses indicated that the assumptions of normality, linearity, and homoscedasticity were satisfied. The Durbin–Watson values (1.911 for Model 1 and 1.891 for Model 2) were close to 2, showing no autocorrelation among the residuals. The cumulative variance explained by the effects of these significant independent variables was approximately 32% (Model 1: adjusted R^2^ = 0.322; Model 2: adjusted R^2^ = 0.327). No multicollinearity was detected among the independent variables in the regression models (Variance Inflation Factor < 10, Tolerance > 0.10).

## 4. Discussion

As life expectancy continues to increase, the quality of life in older adults has become increasingly important. Therefore, significant attention and effort are required from the society to ensure that older adults lead happy and fulfilling lives. While the quality of life of older adults is dictated by a complex interaction of various factors [11], health status, in particular, has a significant impact [47]. Therefore, paying attention to the more prevalent disorders and diseases in older adults will contribute to establishing intervention strategies that can effectively prevent an early decline in the quality of life. In particular, considering recent results showing a relatively high prevalence of swallowing dysfunction even in healthy older adults [23,24], there is a necessity to identify the impact of these disorders on the quality of life. Hence, this pilot cross-sectional study analyzed the association between swallowing dysfunction and quality of life among healthy, community-dwelling older adults who had no conditions known to cause swallowing dysfunction and were cognitively normal on a dementia screening test.

First, the mean quality of life (assessed using CASP-19) score of the participants was approximately 35.76 (±9.94). Because individuals experience various geriatric problems, such as declining physical and mental health and reduced income, in older adulthood, the quality of life should be evaluated using tools specifically designed for this population that reflect the multidimensional nature of life [48]. The CASP-19, employed in this study, has a sufficient number of items to assess the quality of life, places emphasis on the satisfaction of psychological and social needs [49], and is associated with active and successful aging [50]. The participants’ quality of life scores was slightly lower than that reported in previous studies using the same instrument [46,47]. It could be attributable to the higher age of the participants in this study. In addition, because the quality of life varies depending on the diverse characteristics of the participants, such as sex and existing diseases [4,51], direct comparisons with older adults from other cultures may be of limited value. However, despite South Korea’s achievements in industrialization and democratization, the overall life satisfaction and happiness of its citizens remain relatively low [52]. Given that the proportion of older adults in Korea is increasing at the fastest rate in the world, improving the quality of life in older adults is crucial for improving the overall quality of life in Korean society [53]. Further research on the quality of life of older adults is expected to contribute to solving serious problems in aging societies, such as suicide.

Additionally, this study used both the self-reported DRAS and the examiner-administered RSST to identify the risk of swallowing dysfunction. The results showed that approximately 30% (*n* = 32) of the participants had a high risk of swallowing dysfunction assessed using the DRAS, while 74% (*n* = 78) had a high risk assessed using RSST. The notable difference in prevalence between the DRAS and RSST was hypothesized to reflect participants’ limited awareness of mild swallowing dysfunction, as suggested by previous studies [24,31,32], as well as the potential influence of social desirability bias. Although the prevalence rates vary considerably depending on the instrument used, these findings reaffirm the possibility that a substantial number of healthy older adults have swallowing dysfunction. Similarly, approximately one in three independent community-dwelling older adults is suspected to have swallowing dysfunction [23,54,55]. As the physiological process of swallowing naturally changes with age, aging is a risk factor for swallowing dysfunction [18]. Physiological changes are particularly evident in the upper esophageal sphincter and pharyngeal region, which may lead to poor esophageal sensation, causing signs of swallowing dysfunction [56]. Additionally, age-related decline in the strength of the tongue and cheek muscles, both of which are significantly involved in swallowing, can lead to a diminished chewing ability and bolus clearance [57]. Furthermore, tooth loss or lack of dental prostheses can exacerbate the severity of swallowing dysfunction [58]. Therefore, considering the increasing average age of community-dwelling older adults, this study suggests that swallowing dysfunction, which can be accompanied by various discomforts and serious complications, should be included in geriatric health screening programs to monitor its prevalence.

Second, the results of the *t*-tests and ANOVA indicated that older adults in the high-risk groups for swallowing dysfunction had a lower quality of life than did the control group. In the final regression model adjusted for socio-demographic characteristics, health status, and health behavior factors, which was designed to assess the strength of association between quality of life and swallowing dysfunction, the high-risk groups for swallowing dysfunction (DRAS score ≥ 6 and RSST times ≤ 2) showed a lower quality of life than did the control group (β = −0.179 and −0.201, respectively) (*p* < 0.05). To the best of our knowledge, this is one of the few studies to suggest an association between the multidimensional quality of life and swallowing dysfunction among healthy community-dwelling older adults, making it difficult to compare our findings with those of previous studies. However, our findings support the results of a previous study that demonstrated an association between swallowing difficulties and swallowing-related quality of life in community-dwelling older adults, even though the prior study used the Dysphagia Handicap Index to measure the handicapping effect of swallowing dysfunction to assess the quality of life [55]. Accordingly, the findings of this study suggest that swallowing dysfunction can decrease the quality of life in healthy older adults. They also indicated that the level of swallowing dysfunction experienced by healthy older adults can be severe enough to diminish their quality of life. Alternatively, we can also interpret these findings to suggest that healthy active older adults living independently in the community tend to perceive various discomforts or difficulties in swallowing, even when they are mild, as obstacles to a happy and satisfying life, compared to patients with swallowing dysfunction caused by specific conditions. The major signs of swallowing dysfunction reported by the participants were choking or coughing, longer meal durations, and avoidance of certain foods; older adults who experienced these discomforts may not fully enjoy their mealtimes. This discomfort can also discourage older adults from maintaining social relationships and narrowing their social networks, ultimately leading to isolation. Social isolation is directly related to a lower quality of life [52]. Dodderi et al. [55] reported that approximately 30% of community-dwelling older adults experience depression because they cannot eat the foods they desire because of swallowing difficulties. Accordingly, we can infer that eating the desired foods is an emotional pleasure for older adults. Furthermore, prolonged swallowing difficulties in older adults can change in meal patterns [59], and these unwanted dietary changes may diminish the joy of eating, ultimately diminishing the quality of life. Another study showed that reduced oral intake in individuals with swallowing dysfunction was associated with a poor quality of life and suggested that strategies to increase oral intake can improve the quality of life [60]. Eating is essential for survival; however, in the modern society, its meaning and role are expanding. Social eating and drinking, defined as eating and drinking with others, can comprehensively affect well-being by significantly enhancing joy in life [61]. Therefore, swallowing dysfunction can cause various problems, including discomfort such as choking, unwanted dietary changes that diminish the joy of eating, and narrower social networks, which in turn negatively affect social and psychological aspects of life, ultimately lowering the overall quality of life in older adults. Accordingly, our findings suggest that interventions targeting swallowing function should be incorporated into strategies to enhance the quality of life of healthy older adults. Oral problems including tooth loss and oral dryness, which are risk factors for age-related swallowing dysfunction, should be monitored carefully. In addition, awareness regarding the importance of swallowing function in the quality of life of healthy older adults should be promoted among older adults, caregivers, and social workers. Community education is crucial to prevent older adults from misperceiving swallowing dysfunction as a normal part of aging and missing opportunities for treatment and care. Healthcare professionals should provide psychological and emotional support with the early detection and appropriate management of swallowing dysfunction in healthy older adults living in the community.

In addition, this study reaffirmed that older adults who engage in healthy behaviors, such as exercise and tooth brushing, tend to have a higher quality of life. Older adults with a strong sense of purpose in life are more likely to maintain healthy behaviors, participate in disease prevention more actively, and have a higher quality of life [47,62]. Therefore, we emphasize the importance of interventions that support older adults in maintaining a strong sense of purpose in life, including lifelong learning, to improve their quality of life. The final model of this study showed that a higher economic status was associated with a better quality of life, consistent with the results of a previous study that used CASP-12, a shorter form of CASP-19, to examine the quality of life in older adults [48]. However, other studies reported that quality of life is not related to sociodemographic characteristics among older adults [47]. In general, while objective indicators such as income provide useful insights into the quality of life, the more dominant view is that life satisfaction perceived by each individual varies, even under similar objective conditions [52]. Finally, although a positive association was observed between Christianity and quality of life, this result should be interpreted with caution because the number of Christians was small (*n* = 34) and the study did not assess religious activities, which are known to have a stronger association with quality of life than religious affiliation alone [63].

To date, few studies have demonstrated a correlation between swallowing dysfunction, a relatively common condition among healthy older adults, and quality of life from a multidimensional perspective. The significance of this study lies in identifying a potential association between swallowing dysfunction and overall quality of life in older adults, particularly in healthy older adults without any swallowing dysfunction-causing diseases, cognitive impairment, or difficulties in performing daily functions, using the CASP-19 scale, which is specifically designed to measure the quality of life in older adults from a multidimensional perspective.

Despite the potential contributions of this study to the literature, it has several limitations that suggest the need for further research on this topic. First, the participants were selected using convenience sampling, and the small sample size may have limited the generalizability of the findings. Second, because the study was conducted cross-sectionally, it is difficult to determine causal relationships between the variables used, particularly between swallowing dysfunction and quality of life of older adults. Third, the DRAS and RSST, which were used to identify high-risk groups for swallowing dysfunction, are screening tools rather than diagnostic instruments. The DRAS had relatively low sensitivity compared to the 3 oz water test (sensitivity 57%, specificity 70%) [42], while the RSST demonstrates very high specificity but moderate specificity compared to video-fluorography, one of the gold standards for diagnosing swallowing dysfunction (sensitivity 98%, specificity 66%) [44]. Future research should review participants’ medical histories and incorporate additional assessments of swallowing ability, such as video-fluoroscopic swallowing studies, to improve diagnostic accuracy. In addition, the prevalence of swallowing dysfunction observed in this study may not generalize to frailer or medically complex older adults, as individuals with conditions known to cause swallowing dysfunction were excluded. Fourth, although this study controlled for multiple factors that could affect the overall quality of life, more powerful hidden variables may have not been considered. For example, it did not consider important factors that may affect both quality of life and swallowing function in older adults, including chronic diseases, depression, emotional network [64], and the level of purpose in life [47]. Lastly, this study used several self-reported data, including health behaviors and DRAS. However, such self-report surveys can lead to various problems such as recall and social desirability bias. To address this, future studies should combine self-report surveys with objective measures to enhance data validity and minimize biases. Building on our findings, longitudinal studies with larger samples and broader consideration of factors related to quality of life are needed to reduce residual confounding and clarify the impact of swallowing dysfunction on quality of life in older adults.

## 5. Conclusions

This pilot cross-sectional study found that swallowing dysfunction was significantly associated with reduced quality of life in community-dwelling healthy older adults. These findings suggest that the prevalence and risk factors of swallowing dysfunction should be carefully monitored, and that interventions to maintain or improve swallowing function should be integrated into strategies to improve quality of life. As this preliminary study could not establish a causal relationship, the results should be confirmed through longitudinal studies with larger sample sizes.

## Figures and Tables

**Table 1:** Quality of life by socio-demographic characteristics.

Variables	*n*	General Quality of Life	
		Mean ± SD	t, F, or x^2^ (*p*)
Sex			0.686
Male	13	37.54 ± 12.15	(0.494)
Female	92	35.51 ± 9.65	
Age group (years)			1.252
60s	26	38.42 ± 9.13	(0.290)
70s	58	34.98 ± 10.68	
80s	21	34.62 ± 8.53	
Education			2.021
≤Elementary	31	33.19 ± 8.84	(0.116)
Middle school diploma	29	37.17 ± 10.33	
High school diploma	26	34.46 ± 10.53	
≥Junior college	19	39.58 ± 9.43	
Economic status			x^2^ = 9.418
High	40	39.50 ± 8.44 ^a^	(0.009)
Middle	62	33.23 ± 10.32 ^b^	
Low	3	38.33 ± 3.78 ^ab^	
Religion			2.443
None	31	33.71 ± 11.01	(0.069)
Buddhist	26	33.15 ± 11.08	
Christian	34	39.00 ± 7.27	
Catholic	14	37.29 ± 9.38	
Total	105	35.76 ± 9.94	

Analyzed using *t*-test, one-way analysis of variance, or Kruskal–Wallis test. t, t-test statistic; F, F-test statistic; χ^2^, chi-square statistic (Kruskal–Wallis test). All values are expressed as means ± SDs. ^a,b^ Same characters denote no significant differences assessed using Scheffe’s multiple comparison at α = 0.05 or using the Mann–Whitney U test (with Bonferroni correction). SD, standard deviation.

**Table 2:** Quality of life by health behaviors.

Variables	*n*	General Quality of Life	
		Mean ± SD	t, F, or x^2^ (*p*)
Health check-up in past 2 years			1.398
Yes	93	36.25 ± 10.01	(0.165)
No	12	32.00 ± 8.94	
Smoking status			x^2^ = 0.095
Current smoker	3	35.00 ± 9.84	(0.954)
Former smoker	9	34.11 ± 12.86	
Never smoker	93	35.95 ± 9.75	
Alcohol consumption (current)			−1.611
Yes	12	40.08 ± 9.72	(0.110)
No	93	35.20 ± 9.89	
High-intensity exercise (≥10 min per day)			0.674
Yes	23	37.00 ± 10.94	(0.502)
No	82	35.41 ± 9.69	
Moderate-intensity exercise (≥10 min per day)			2.205
Yes	54	37.80 ± 10.48	(0.030)
No	51	33.61 ± 8.94	
Frequency of daily toothbrushing			−2.731
0–2 times	46	32.85 ± 11.08	(0.007)
≥3 times	59	38.03 ± 8.37	
Periodic dental check-ups			2.090
Yes	73	37.04 ± 10.05	(0.041)
No	32	32.84 ± 9.20	
Periodic dental scaling			2.826
Yes	73	37.52 ± 9.63	(0.006)
No	32	31.75 ± 9.63	
Tongue cleaning			x^2^ = 1.957
Always (every day)	68	36.59 ± 10.14	(0.376)
Sometimes (every 1 or 2 weeks)	30	33.94 ± 9.90	
Never	7	35.43 ± 8.26	
Use of interdental cleaning devices			0.310
Always (every day)	37	36.19 ± 10.88	(0.734)
Sometimes (every 1 or 2 weeks)	39	39.28 ± 8.56	
Never	29	34.52 ± 10.65	

Analyzed using *t*-test, one-way analysis of variance, or Kruskal–Wallis test. t, *t*-test statistic; F, F-test statistic; χ^2^, chi-square statistic (Kruskal–Wallis test). All values are expressed as means ± SDs. SD, standard deviation.

**Table 3:** Quality of life by oral and general health status.

Variables	*n*	General Quality of Life	
		Mean ± SD	t, F, or x^2^ (*p*)
Self-perceived general health status			x^2^ = 10.166
Poor	9	33.67 ± 7.43 ^ab^	(0.006)
Fair	54	33.11 ± 10.77 ^a^	
Good	42	39.62 ± 8.05 ^b^	
Number of systemic diseases			2.228
0–1	33	38.91 ± 9.83	(0.030)
≥2	72	34.32 ± 9.73	
Self-perceived stress status			x^2^ = 13.865
Very low	37	39.84 ± 8.92 ^a^	(0.003)
Low	46	33.87 ± 10.23 ^a^	
High	19	34.74 ± 8.20 ^a^	
Very high	3	21.00 ± 5.00 ^b^	
Frailty status			x^2^ = 6.416
Robust	59	36.90 ± 9.70 ^a^	(0.040)
Pre-frailty	41	35.32 ± 10.24 ^ab^	
Frailty	5	26.00 ± 4.24 ^b^	
History of toothache			−0.971
Yes	35	34.43 ± 10.60	(0.334)
No	70	36.43 ± 9.61	
Masticatory discomfort			0.742
Yes	19	33.79 ± 10.90	(0.479)
Slight	18	34.61 ± 11.24	
No	68	36.62 ± 9.35	
Number of remaining teeth			−2.732
0–25	29	31.52 ± 9.95	(0.009)
≥26	76	37.38 ± 9.52	
Use of dentures			1.352
No	91	36.27 ± 9.99	(0.179)
Yes	14	32.43 ± 9.29	
Need for dental prostheses (maxillary)			2.247
No	61	37.57 ± 9.84	(0.027)
Yes	44	33.25 ± 9.64	
Need for dental prostheses (mandibular)			1.772
No	64	37.13 ± 9.78	(0.079)
Yes	41	33.63 ± 9.94	
Chewing ability (score)			4.543
6–7	15	31.93 ± 11.46 ^a^	(0.013)
8	63	34.73 ± 9.73 ^ab^	
9–10	27	40.30 ± 8.17 ^b^	
Dysphagia risk (DRAS)			2.483
Normal (score ≤ 5)	73	37.38 ± 9.35	(0.016)
High risk (score ≥ 6)	32	32.06 ± 10.42	
Dysphagia risk (RSST)			2.494
Normal (≥3 times)	27	39.78 ± 9.09	(0.014)
High risk (≤2 times)	78	34.37 ± 9.90	

Analyzed using *t*-test, one-way analysis of variance, or Kruskal–Wallis test. t, t-test statistic; F, F-test statistic; χ^2^, chi-square statistic (Kruskal–Wallis test). All values are expressed as means ± SDs. ^a,b^ Same characters denote no significant differences assessed using Scheffe’s multiple comparison at α = 0.05 or using the Mann–Whitney U test (with Bonferroni correction). SD, standard deviation; DRAS, Dysphagia Risk Assessment Scale; RSST, Repetitive Saliva Swallowing Test.

**Table 4:** Intensity of association between swallowing dysfunction risk and quality of life.

Division	Variables	β	t	*p-*Value *	VIF
	Economic status	0.240	2.727	0.008	1.185
	Religion = Christian (ref. Non-Christian)	0.226	2.715	0.008	1.062
	Moderate-intensity exercise (No) (ref. Yes)	−0.190	−2.323	0.022	1.030
Model 1	Frequency of daily toothbrushing	0.223	2.659	0.009	1.074
	Periodic scaling (No) (ref. Yes)	−0.187	−2.233	0.028	1.073
	Self-perceived stress levels	−0.206	−2.397	0.018	1.134
	High-risk group for swallowing dysfunction (DRAS score ≥6)	−0.179	−2.171	0.032	1.038
	F = 8.048 (<0.001), adj. R^2^ = 0.322, Durbin-Watson = 1.911				
	Economic status	0.237	2.698	0.008	1.188
	Religion = Christian (ref. Non-Christian)	0.256	3.055	0.003	1.081
	Moderate-intensity exercise (No) (ref. Yes)	−0.218	−2.613	0.010	1.071
Model 2	Frequency of daily toothbrushing	0.190	2.228	0.028	1.126
	Periodic scaling (No) (ref. Yes)	−0.185	−2.219	0.029	1.073
	Self-perceived stress levels	−0.199	−2.311	0.023	1.142
	High-risk group for swallowing dysfunction (RSST times ≤ 2)	−0.201	−2.331	0.022	1.147
	F = 8.203 (<0.001), adjusted R^2^ = 0.327, Durbin-Watson = 1.891				

* Stepwise multiple linear regression analysis. Models 1 and 2 were adjusted for sex, age, education, economic status, religion, periodic dental checkups, general health status, number of systemic diseases, frailty status, number of remaining teeth, need for maxillary or mandibular prosthesis, and chewing ability. Model 2: High-risk group for swallowing dysfunction (DRAS score ≥ 6) used in Model 1 was replaced with high-risk group for swallowing dysfunction (RSST times ≤ 2). VIF, Variance Inflation Factor; DRAS, Dysphagia Risk Assessment Scale; RSST, Repetitive Saliva Swallowing Test.

## Data Availability

The data presented in this study are available on request from the corresponding author due to privacy restrictions.

## Abbreviations

The following abbreviations are used in this manuscript:

WHO	World Health Organization
MMSE-K	Mini-Mental State Examination-Korean version
ADL-K	Korean Activities of Daily Living
DRAS	Dysphagia Risk Assessment Scale
RSST	Repetitive Saliva Swallowing Test
ANOVA	Analysis of variance
SD	standard deviation
VIF	variance inflation factor
